# First report on C-banding, fluorochrome staining and NOR location in holocentric chromosomes of *Elasmolomus* (*Aphanus*) *sordidus* Fabricius, 1787 (Heteroptera, Rhyparochromidae)

**DOI:** 10.3897/zookeys.319.4265

**Published:** 2013-07-30

**Authors:** Vikas Suman, Harbhajan Kaur

**Affiliations:** 1 Department of Entomology, Y.S. Parmar University of Horticulture and Forestry, Nauni, Solan 173 230, Himachal Pradesh, India; 2 Department of Zoology and Environmental Sciences, Punjabi University, Patiala 147 002, Punjab, India

**Keywords:** C-banding, DAPI, CMA_3_, NOR location

## Abstract

In spite of various cytogenetic works on suborder Heteroptera, the chromosome organization, function and its evolution in this group is far from being fully understood. Cytologically, the family Rhyparochromidae constitutes a heterogeneous group differing in chromosome numbers. This family possesses XY sex mechanism in the majority of the species with few exceptions. In the present work, multiple banding techniques viz., C-banding, base-specific fluorochromes (DAPI/CMA_3_) and silver nitrate staining have been used to cytologically characterize the chromosomes of the seed plant pest *Elasmolomus (Aphanus) sordidus* Fabricius, 1787 having 2n=12=8A+2m+XY. One pair of the autosomes was large while three others were of almost equal size. At diplotene, C-banding technique revealed, that three autosomal bivalents show terminal constitutive heterochromatic bands while one medium sized bivalent was euchromatic. Microchromosomes (m-chromosomes) were positively heteropycnotic. After DAPI and CMA_3_ staining, all the autosomal bivalents showed equal fluorescence, except CMA_3_ positive signals, observed at both telomeric heterochromatic regions of one medium sized autosomal bivalent. Silver nitrate staining further revealed that this chromosome pair carries Nucleolar Organizer Regions (NORs) at the location of CMA_3_ positive signals. The X chromosome showed a thick C-band, positive to both DAPI /CMA_3_ while Y, otherwise C-negative, was weakly positive to DAPI and negative to CMA_3_, m-chromosomes were DAPI bright and CMA_3_ dull.

## Introduction

Heteroptera is a large cosmopolitan suborder comprising about 42,300 known species (Henry 2009). The species of Heteroptera are distributed into 7 infraorders and a total of 24 superfamilies worldwide (Schuh and Slater 1995). Lygaeidae, Rhyparochromidae Pyrrhocoridae, Coreidae, Pentatomidae, Reduviidae and Miridae are some of the major families, each having its individual economic importance (Schaefer and Panizzi 2000). Rhyparochromidae (seed bugs) were considered by most workers to be a subfamily within the Lygaeidae until revision by Henry (1997) who recognized them at the family level. Rhyparochromids are mostly ground dwellers, liveing in the shadow vegetation and feeding primarily on seeds. *Elasmolomus (Aphanus) sordidus* is a serious pest, occurring on pods left drying in the fields and in stores. Groundnuts and sesame pods infested by this insect have shrivelled kernels. Like other heteropterans, Rhyparochromidae are characterized by holokinetic chromosomes and post reductional division of sex chromosomes, as well by presence of m-chromosomes and XY sex mechanism in all the species with few exceptions (Ueshima 1979).

In the present contribution, cytological characterization of *Elasmolomus (Aphanus) sordidus*, reported as *Aphanus sordidus* having chromosomal complement 2n=12=8A+2m+XY (Parshad 1957), has been done using different banding techniques. The amount and location (C-banding) and composition (AT/GC base richness) of heterochromatin have been studied. Further silver banding was employed to locate the position and number of nucleolar organiser regions (NORs). The application of CMA_3_/DAPI banding revealed correspondence between NORs (r-DNA sites) and GC rich domains.

## Material and methods

Adult males of *Elasmolomus sordidus* (9 specimens) were collected from fields of sesame and groundnut plants in Punjab (India). Insects were dissected to remove the gonads and air dried slides were prepared. Aged air dried slides were used for C-banding after Kaur et al. (2010). To study the localization of NORs, silver staining was done using one step method with a protective colloidal developer (gelatine and formic acid) (Howell and Black 1980). To reveal the base composition of C-heterochromatin, two fluorochromes: AT sequence specific DAPI (4-6’ Diamidino-2-phenylindole) and GC sequence specific CMA_3_ (chromomycin A_3_), were applied, following the protocol suggested by Manicardi and Gautam (1994). Well-spread stages were photographed under the microscope Nikon-Optiphot-2. Slides stained with fluorochrome dyes DAPI/CMA_3_ were studied and photographed under Nikon fluorescent microscope using UV filter (for DAPI) and BV (for CMA_3_).

## Results

The chromosomal complement consisted of twelve elements. Of these, eight were autosomes, two were m-chromosomes, while two of different sizes were sex chromosomes, large X and small Y, respectively. The chromosomal complement was confirmed as 2n=12=8A+2m+XY.

### C-banding

**Figure 1–8.**
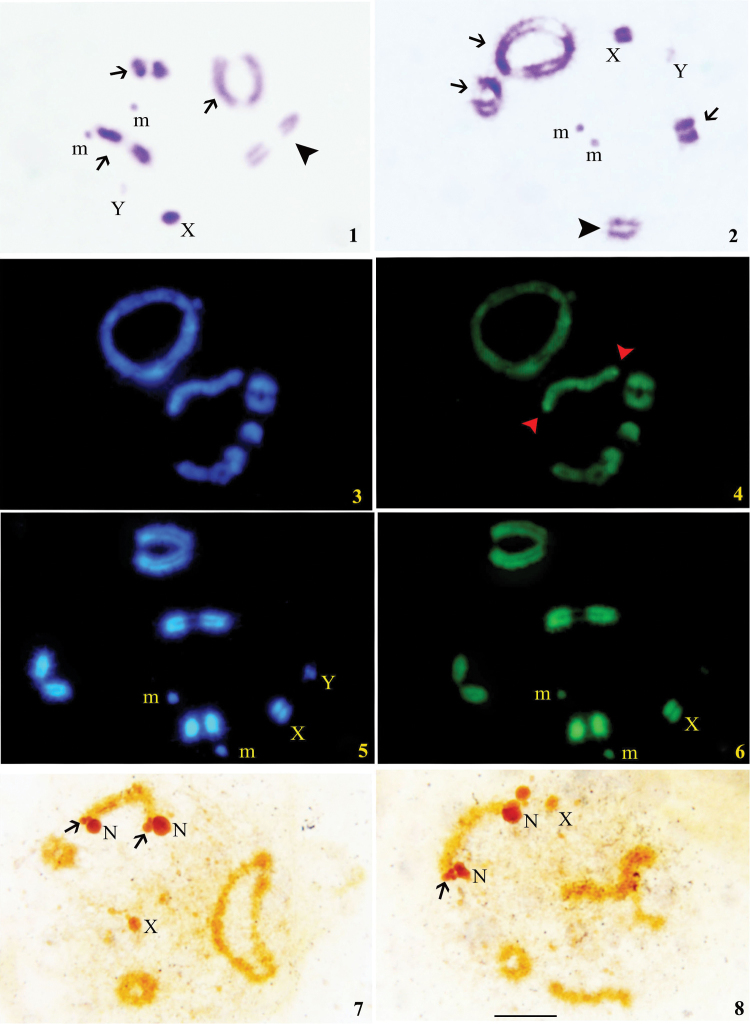
**C-banding (1, 2) 1, 2** Diplotene stages showing distribution of C-bands. Arrows showing heterochromatic chromosomes while arrowhead showing single euchromatic chromosome. **Sequence-specific banding (3–6) 3** Diplotene stage with DAPI **4** Diplotene stage with localized CMA_3_ signals on one autosomal bivalent (shown by arrows) **5** Late diplotene stage with DAPI **6** Late diplotene stage with CMA_3_. **Silver banding (7, 8) 7, 8** Diplotene stages showing location of NORs (shown by arrows) and nucleolar bodies (N). Bar=0.01 mm.

At diplotene, three bivalents showed terminal C-bands while one was euchromatic. The X chromosome showed thick C-band covering almost two thirds of the chromosome while Y was C-negative; m-chromosomes were slightly C-positive (Figs 1, 2).

### DAPI/CMA_3_ staining

All the four autosomal bivalents showed equal fluorescence with both DAPI and CMA_3_ (Figs 3, 4). However, one of the medium sized autosomal bivalents showed bright CMA_3_ signals at both ends, which correspond to NORs (Fig. 4). The X was positive to both DAPI/CMA_3_ while Y was weak to DAPI and negative to CMA_3_; m-chromosomes were DAPI bright and CMA_3_ dull (Figs 5, 6).

### Silver staining

NORs were found to be associated with both ends of a medium-sized autosomal bivalent (Figs 7, 8).

## Discussion

*Elasmolomus sordidus* is a pest of pod crops, mainly groundnut and sesame in India. Parshad (1957) was first to study its standard chromosomal complement (2n=12=8A+2m+XY) and male meiosis of this species (as *Aphanus sordidus*). The same chromosomal complement has been observed by the present authors. In the present study, C-banding, silver staining and DNA sequence-specific staining have been used to reveal the distribution and constitution of constitutive heterochromatin and also to find the correspondence between NORs and GC-rich regions.

### C-banding

Terminal C-bands have been observed in three autosomal pairs of *Elasmolomus sordidus*. In Heteroptera, the terminal C-bands are of wide occurrence. This kind of C-band location has been reported in *Antiteuchus mixtus* (Fabricius, 1787) (Pentatomidae) by Lanzone and Souza (2006), in *Dieuches uniguttatus* (Thumb, 1822) and *Dieuches insignis* (Distant, 1918) (Rhyparochromidae) by Kaur et al. (2010). Usually, telomeric bands are absent, if interstitial blocks are present in a chromosome. This is reported in one or two chromosomes of *Nezara viridula* Linnaeus, 1758 (Pentatomidae) and *Triatoma patagonica* Del-Ponte, 1929 (Reduviidae) by Camacho et al. (1985) and Panzera et al. (1997) respectively.

One of the autosomal bivalent in *Elasmolomus sordidus* was found to be euchromatic. A similar condition is observed in *Nezara icterica* (Horvath, 1889) (Pentatomidae) by Dey and Wangdi (1990), in *Dieuches coloratus* Distant, 1909 (Rhyparochromidae) by Kaur et al. (2010) and in *Neophysopelta schlanbuschi* Ahmad & Abbas, 1987 (Largidae) by Suman et al. (2012).

The X chromosome is almost (2/3) completely C-positive and this condition has been earlier reported in Pentatomidae by Camacho et al. (1985), in Tingidae by Grozeva and Nokkala (2001) and in Nabidae by Grozeva et al. (2004), whereas, the Y chromosome, is C-negative. This condition is not uncommon in Heteroptera and has been reported previously in some species belonging to Coreidae, Pentatomidae and Tingidae (Muramoto 1980, Camacho et al. 1985, Dey and Wangdi 1990, Grozeva and Nokkala 2001).

Microchromosomes were originally described by Wilson (1905); since then they have been discovered in many heteropteran families, including Rhyparochromidae. Microchromosomes are C-positive in *Elasmolomus sordidus*. Similar observation have been made in *Leptoglossus impictus* (Stål, 1860) and *Phthia picta* (Drury, 1773) (Coreidae) by Bressa et al. (2005) and in *Dieuches uniguttatus* and *Dieuches insignis* (Rhyparochromidae)by Kaur et al. (2010). Microchromosomes are DAPI bright and CMA_3_ dull. Similar set of observations have been previously made by Kaur et al. (2010) in *Dieuches uniguttatus* and *Dieuches insignis* (Rhyparochromidae). Information on chromatin composition of m-chromosomes is still very poor and their genetic constitution is not fully known.

### DAPI/CMA_3_ staining

The use of DNA binding fluorochromes having different base specificities allows a better characterization of heterochromatic regions in terms of their relative enrichment with AT or GC base pairs. In Heteroptera, still there is little information on heterochromatin base composition. The bright fluorescence after DAPI and CMA_3_ staining observed in *Elasmolomus sordidus* indicates that the constitutive heterochromatic regions possess interspersed AT and GC repeats. Similar observations have been made in *Edessa meditabunda* (Fabricius, 1974) and *Edessa rufomarginata* (De Geer, 1773) (Pentatomidae) by Rebagliati et al. (2003), in *Antiteuchus mixtus*, *Antiteuchus macraspis* (Perty, 1834), *Antiteuchus sepulcralis* (Fabricius, 1803) (Pentatomidae) by Lanzone and Souza (2006) and in *Arachnocoris trinitatus* Bergroth, 1916 (Nabidae) by Kuznetsova et al. (2007).

After silver banding and fluorochrome staining, the localization of CMA_3_ positive bands in NOR regions on medium sized autosomal bivalent was revealed. It was confirmed that ribosomal genes are GC rich. This correspondence of CMA_3_ signals with NORs have also been reported for several true bug species at interstitial or terminal positions either on autosomes or sex chromosomes by Gonzalez-Garcia et al. (1996), Papeschi et al. (2003), Rebagliati et al. (2003), and Grozeva et al. (2004). However, NORs do not always show GC base richness as is reported in *Carlisis wahlbergi* Stål, 1858 (Coreidae) by Fossey and Liebenberg (1995).

A common feature of the sex chromosomes of Heteroptera is that they demonstrate bright fluorescence after both DAPI and CMA_3_ during the meiotic prophase (Rebagliati et al. 2003). In the present study, the X chromosome showed fluorescence after both DAPI and CMA_3_. Similar observations have been also made in *Cimex emarginatus* Simov, Ivanova & Schunger, 2006 by Grozeva and Nokkala (2002), *Cimex lectularius* (Cimicidae) by Grozeva et al. (2010), in *Edessa meditabunda* and *Edessa rufomarginata* (Pentatomidae) by Rebagliati et al. (2003), in *Athaumastus haematicus* (Stål, 1860), *Leptoglossus impictus* and *Phthia picta* (Coreidae), *Jadera sanguinolenta* (Fabricius, 1775) (Rhopalidae) by Bressa et al. (2005), in *Antiteuchus mixtus*, *Antiteuchus macraspis* and *Antiteuchus sepulcralis* (Pentatomidae) by Lanzone and Souza (2006). In the present study, however, the Y chromosome is C-negative, but DAPI positive and CMA_3_ negative. Similar observations have been made in *Triatoma vitticeps* (Stål, 1859) (Reduviidae) by Severi-Aguiar et al. (2006).

### Silver staining

The silver impregnation stains not only the NORs but also the nucleolus at specific points of some chromosomes (Castanhole et al. 2008). In the present study, NORs were found to present on terminal regions of one of medium sized autosomal pairs of *Elasmolomus sordidus*, like in *Nysius californicus* Stål, 1859 (Lygaeidae) (Souza et al. 2007), and in *Arachnocoris trinitatus* Bergroth, 1916 (Nabidae) (Kuznetsova et al. 2007). However, in Belostomatidae, NORs have been reported on either autosomes, on sex chromosomes or on both autosomes and sex chromosomes (Papeschi and Bidau 1985).

## Conclusion

Till date, very few Rhyparochromid species have been analysed cytologically based on banding techniques. The present study was able to reveal some cytogenetic characters which were used as markers for better knowledge of chromosome organization and the identification of separate chromosomes in *Elasmolomus sordidus*. Much more information about true bug chromosomes could be obtained if new molecular cytogenetic techniques involving FISH (fluroscence *in situ* hybridization) mapping of chromosomes are used (Grozeva et al. 2011 and references therein, Kuznetsova et al. 2012 and references therein).
